# Supplementary material for: Inductive detection of influence operations via graph learning
Source: Sci Rep. 2023 Dec 19;13:22571. doi: 10.1038/s41598-023-49676-z (PMC10730915; doi:10.1038/s41598-023-49676-z)
Supplement: Supplementary file 1 — Supplementary Information. [file 41598_2023_49676_MOESM1_ESM.pdf]

# Supplementary Information

## A Additional CDF distance metrics

Here we show additional metrics for the distance between campaigns shown in Figure 4F. We see that these demonstrate a similar pattern as the Wasserstein distance $W_1$ (area between CDFs) shown in 4f, though the mean squared distance demonstrates stronger clustering.

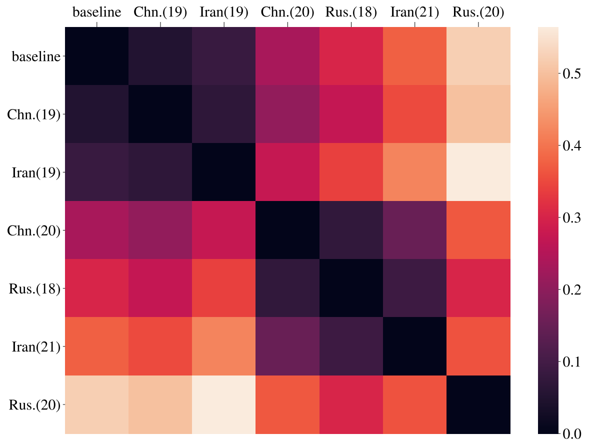


**(a)** Kolmogorov-Smirnov distance between each CDF in Figure 4e.

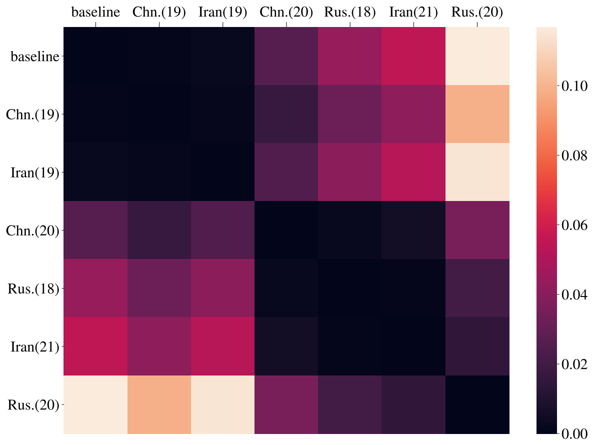


**(b)** Mean squared distance between each CDF in Figure 4e.

**Figure 6.** Additional distance metrics between co-URL distributions. All metrics consistently demonstrate the lagged adoption of co-URLs by Chinese and Iranian campaigns relative to Russian campaigns.

## B Subtask F1(val/test) and AUC(test) for varying censorship thresholds

We show here individual results for F1(val), F1(test), and AUC(test). In the top two panels of each figure, we show the aggregated results of Figure 3, and in the lower six panels we show the subtask results from which these are computed (as the harmonic mean).

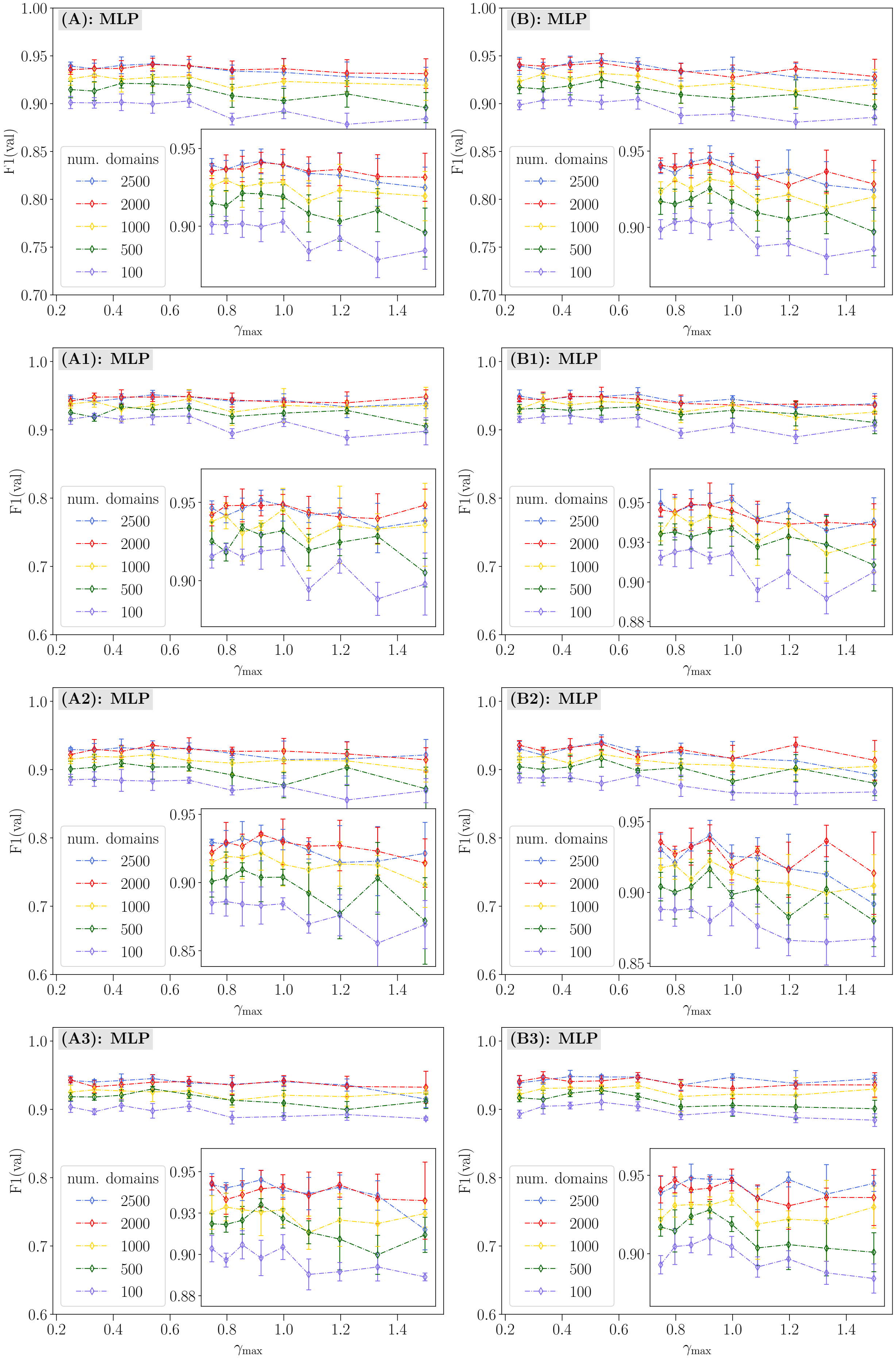


**Figure 7.** F1(val) for aggregated tasks (top two panels) and subtasks (bottom six panels). **Inset:** Series replotted with a rescaled $y$-axis.

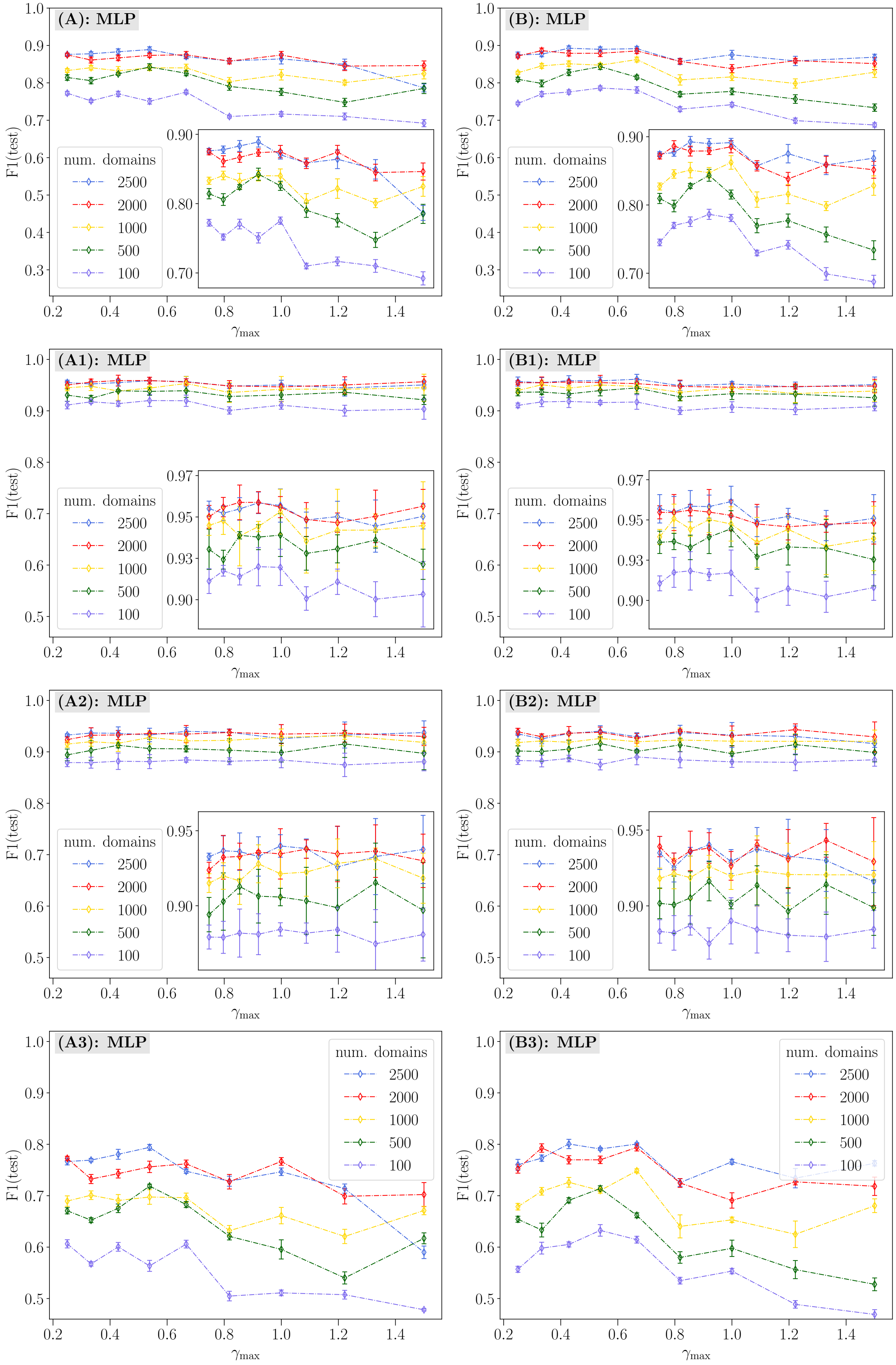


**Figure 8.** F1(test) for aggregated tasks (top two panels) and subtasks (bottom six panels). **Inset:** Series replotted with a rescaled $y$-axis.

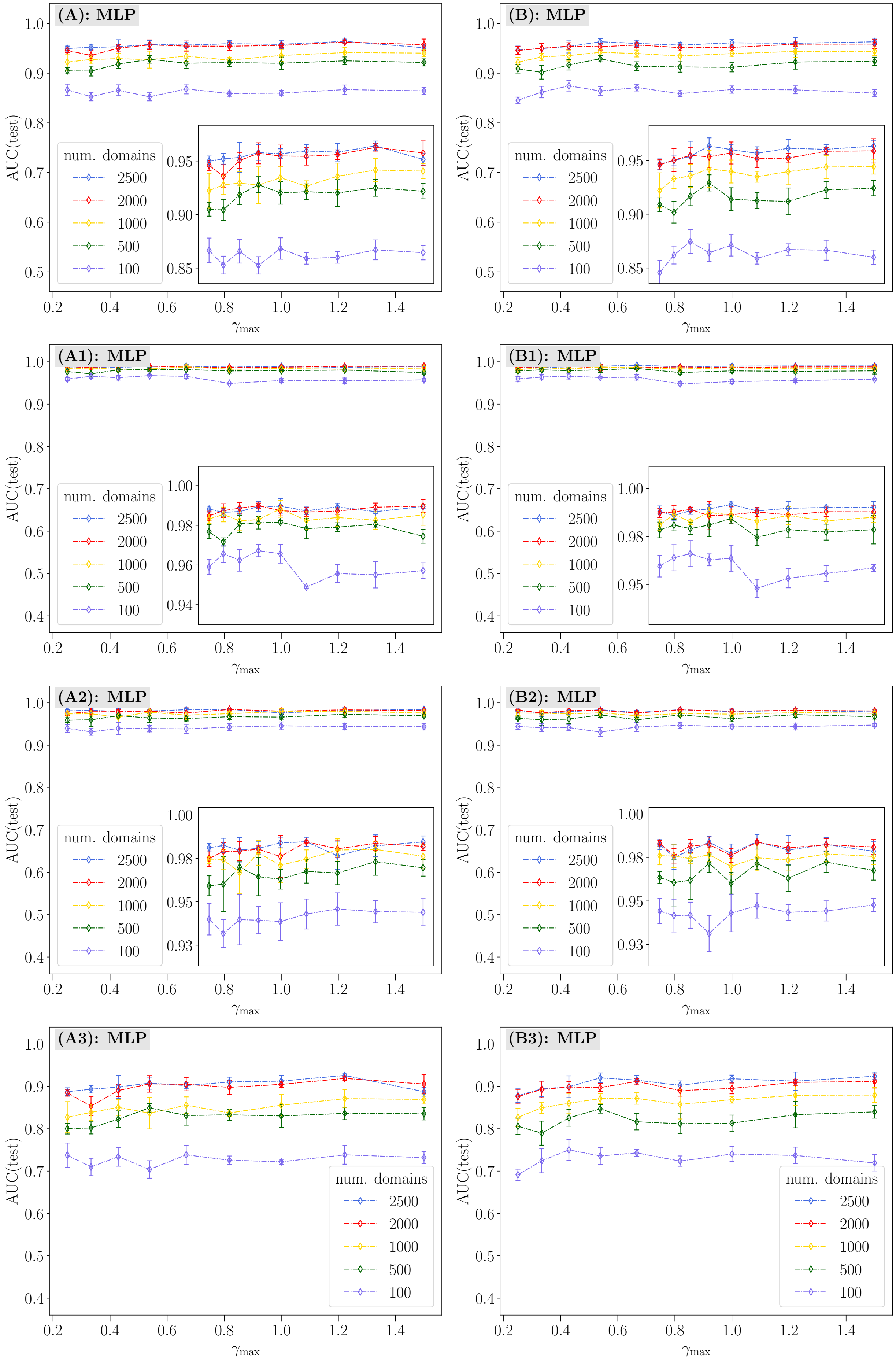


**Figure 9.** AUC(test) for aggregated tasks (top two panels) and subtasks (bottom six panels). **Inset:** Series replotted with a rescaled $y$-axis.

## C Subtask level integrated gradients

In Table 3, we show the mean IG values over subtasks. Here we show explicit results for each of these subtasks. The subtask level results are largely consistent with the aggregated results, although attributions for individual features can vary by up to ±20% across subtasks.

**(A1): Rus(18) → Rus(18) / Rus(20)**

| feature | IG(val.) | IG(test) | IG(base.) |
|---|---|---|---|
| **domains** | $9.44 \times 10^{-2}$ | $5.21 \times 10^{-2}$ | $4.61 \times 10^{-1}$ |
| **node2vec** | $1.54 \times 10^{-1}$ | $3.40 \times 10^{-1}$ | $1.98 \times 10^{-1}$ |
| **LE** | $8.87 \times 10^{-5}$ | $1.26 \times 10^{-4}$ | $4.16 \times 10^{-5}$ |
| **RWPE** | $7.44 \times 10^{-2}$ | $1.77 \times 10^{-1}$ | $3.23 \times 10^{-1}$ |
| **NF** | $6.43 \times 10^{-1}$ | $7.09 \times 10^{-1}$ | $8.11 \times 10^{-1}$ |
| degree | $3.55 \times 10^{-1}$ | $3.44 \times 10^{-1}$ | $2.66 \times 10^{-1}$ |
| cluster. coef. | $1.70 \times 10^{-2}$ | $2.56 \times 10^{-2}$ | $3.34 \times 10^{-2}$ |
| betweenness | $1.58 \times 10^{-2}$ | $2.97 \times 10^{-3}$ | $7.28 \times 10^{-2}$ |
| pagerank | $1.32 \times 10^{-1}$ | $1.78 \times 10^{-1}$ | $3.44 \times 10^{-1}$ |
| HITS | $1.23 \times 10^{-1}$ | $1.58 \times 10^{-1}$ | $9.43 \times 10^{-2}$ |

**(B1): Chn(19) + Iran(19) → Rus(18) / Rus(20)**

| feature | IG(val.) | IG(test) | IG(base.) |
|---|---|---|---|
| **domains** | $9.79 \times 10^{-2}$ | $4.84 \times 10^{-2}$ | $4.78 \times 10^{-1}$ |
| **node2vec** | $1.51 \times 10^{-1}$ | $3.39 \times 10^{-1}$ | $2.09 \times 10^{-1}$ |
| **LE** | $1.04 \times 10^{-4}$ | $1.31 \times 10^{-4}$ | $4.93 \times 10^{-5}$ |
| **RWPE** | $7.26 \times 10^{-2}$ | $1.74 \times 10^{-1}$ | $3.44 \times 10^{-1}$ |
| **NF** | $5.85 \times 10^{-1}$ | $6.40 \times 10^{-1}$ | $8.18 \times 10^{-1}$ |
| degree | $3.54 \times 10^{-1}$ | $3.45 \times 10^{-1}$ | $2.81 \times 10^{-1}$ |
| cluster. coef. | $4.64 \times 10^{-3}$ | $3.48 \times 10^{-3}$ | $9.27 \times 10^{-3}$ |
| betweenness | $9.28 \times 10^{-3}$ | $5.13 \times 10^{-3}$ | $8.90 \times 10^{-2}$ |
| pagerank | $1.19 \times 10^{-1}$ | $1.66 \times 10^{-1}$ | $3.70 \times 10^{-1}$ |
| HITS | $9.81 \times 10^{-2}$ | $1.21 \times 10^{-1}$ | $6.91 \times 10^{-2}$ |

**(A2): Chn(19) → Chn(19) / Chn(20)**

| feature | IG(val.) | IG(test) | IG(base.) |
|---|---|---|---|
| **domains** | $8.67 \times 10^{-2}$ | $1.23 \times 10^{-2}$ | $4.60 \times 10^{-1}$ |
| **node2vec** | $2.33 \times 10^{-1}$ | $2.76 \times 10^{-1}$ | $1.91 \times 10^{-1}$ |
| **LE** | $8.81 \times 10^{-5}$ | $2.71 \times 10^{-4}$ | $3.62 \times 10^{-5}$ |
| **RWPE** | $1.37 \times 10^{-1}$ | $2.97 \times 10^{-1}$ | $3.17 \times 10^{-1}$ |
| **NF** | $7.04 \times 10^{-1}$ | $7.74 \times 10^{-1}$ | $8.03 \times 10^{-1}$ |
| degree | $3.53 \times 10^{-1}$ | $2.45 \times 10^{-1}$ | $2.62 \times 10^{-1}$ |
| cluster. coef. | $6.56 \times 10^{-3}$ | $1.86 \times 10^{-2}$ | $6.04 \times 10^{-3}$ |
| betweenness | $1.68 \times 10^{-3}$ | $3.41 \times 10^{-2}$ | $7.96 \times 10^{-2}$ |
| pagerank | $1.86 \times 10^{-1}$ | $2.73 \times 10^{-1}$ | $3.79 \times 10^{-1}$ |
| HITS | $1.57 \times 10^{-1}$ | $2.04 \times 10^{-1}$ | $7.74 \times 10^{-2}$ |

**(B2): Rus(18) + Iran(19) → Chn(19) / Chn(20)**

| feature | IG(val.) | IG(test) | IG(base.) |
|---|---|---|---|
| **domains** | $5.05 \times 10^{-2}$ | $1.38 \times 10^{-2}$ | $4.21 \times 10^{-1}$ |
| **node2vec** | $2.06 \times 10^{-1}$ | $3.13 \times 10^{-1}$ | $1.73 \times 10^{-1}$ |
| **LE** | $9.03 \times 10^{-5}$ | $3.42 \times 10^{-4}$ | $4.14 \times 10^{-5}$ |
| **RWPE** | $1.18 \times 10^{-1}$ | $3.05 \times 10^{-1}$ | $3.03 \times 10^{-1}$ |
| **NF** | $6.11 \times 10^{-1}$ | $8.58 \times 10^{-1}$ | $7.51 \times 10^{-1}$ |
| degree | $3.05 \times 10^{-1}$ | $2.78 \times 10^{-1}$ | $2.54 \times 10^{-1}$ |
| cluster. coef. | $5.05 \times 10^{-3}$ | $1.36 \times 10^{-2}$ | $7.76 \times 10^{-3}$ |
| betweenness | $9.21 \times 10^{-3}$ | $4.90 \times 10^{-2}$ | $7.45 \times 10^{-2}$ |
| pagerank | $1.65 \times 10^{-1}$ | $3.09 \times 10^{-1}$ | $3.53 \times 10^{-1}$ |
| HITS | $1.27 \times 10^{-1}$ | $2.09 \times 10^{-1}$ | $6.25 \times 10^{-2}$ |

**(A3): Iran(19) → Iran(19) / Iran(21)**

| feature | IG(val.) | IG(test) | IG(base.) |
|---|---|---|---|
| **domains** | $9.86 \times 10^{-2}$ | $2.55 \times 10^{-1}$ | $4.34 \times 10^{-1}$ |
| **node2vec** | $2.47 \times 10^{-1}$ | $4.10 \times 10^{-1}$ | $1.71 \times 10^{-1}$ |
| **LE** | $1.09 \times 10^{-4}$ | $1.20 \times 10^{-3}$ | $3.98 \times 10^{-5}$ |
| **RWPE** | $1.44 \times 10^{-1}$ | $3.76 \times 10^{-1}$ | $3.00 \times 10^{-1}$ |
| **NF** | $7.54 \times 10^{-1}$ | $1.12 \times 10^{0}$ | $7.57 \times 10^{-1}$ |
| degree | $3.79 \times 10^{-1}$ | $3.18 \times 10^{-1}$ | $2.52 \times 10^{-1}$ |
| cluster. coef. | $4.97 \times 10^{-3}$ | $2.32 \times 10^{-3}$ | $5.35 \times 10^{-3}$ |
| betweenness | $9.96 \times 10^{-3}$ | $9.00 \times 10^{-2}$ | $8.25 \times 10^{-2}$ |
| pagerank | $2.06 \times 10^{-1}$ | $4.09 \times 10^{-1}$ | $3.57 \times 10^{-1}$ |
| HITS | $1.55 \times 10^{-1}$ | $2.97 \times 10^{-1}$ | $6.06 \times 10^{-2}$ |

**(B3): Chn(19) + Rus(18) → Iran(19) / Iran(21)**

| feature | IG(val.) | IG(test) | IG(base.) |
|---|---|---|---|
| **domains** | $8.67 \times 10^{-2}$ | $2.17 \times 10^{-1}$ | $4.51 \times 10^{-1}$ |
| **node2vec** | $2.25 \times 10^{-1}$ | $3.61 \times 10^{-1}$ | $1.97 \times 10^{-1}$ |
| **LE** | $1.05 \times 10^{-4}$ | $1.08 \times 10^{-3}$ | $4.88 \times 10^{-5}$ |
| **RWPE** | $1.55 \times 10^{-1}$ | $3.83 \times 10^{-1}$ | $3.33 \times 10^{-1}$ |
| **NF** | $7.18 \times 10^{-1}$ | $1.01 \times 10^{0}$ | $7.83 \times 10^{-1}$ |
| degree | $3.33 \times 10^{-1}$ | $2.69 \times 10^{-1}$ | $2.60 \times 10^{-1}$ |
| cluster. coef. | $5.06 \times 10^{-3}$ | $6.10 \times 10^{-4}$ | $4.63 \times 10^{-3}$ |
| betweenness | $7.53 \times 10^{-3}$ | $6.51 \times 10^{-2}$ | $7.88 \times 10^{-2}$ |
| pagerank | $1.91 \times 10^{-1}$ | $3.47 \times 10^{-1}$ | $3.58 \times 10^{-1}$ |
| HITS | $1.81 \times 10^{-1}$ | $3.32 \times 10^{-1}$ | $8.14 \times 10^{-2}$ |

**Table 4.** IG values of individual subtasks.

## D Integrated gradients by domain

Here we show the most significant domain level integrated gradients. We leave these values signed since they are largely consistent across data splits and subtasks. The signs do not necessarily indicate with certainty whether a feature was used to make a positive ($y = 1$) or negative ($y = 0$) prediction, though one expects a reasonable correspondence.

**(A1): Rus(18) $\rightarrow$ Rus(18) / Rus(20)**

| domain | IG(val.) | domain | IG(test) | domain | IG(base.) |
|---|---|---|---|---|---|
| untappd.com | $4.65 \times 10^{-3}$ | inc.com | $-8.51 \times 10^{-3}$ | nytimes.com | $-2.08 \times 10^{-2}$ |
| senate.gov | $3.80 \times 10^{-3}$ | soundcloud.com | $5.06 \times 10^{-3}$ | abouthub.info | $-1.30 \times 10^{-2}$ |
| nbcnews.com | $2.35 \times 10^{-3}$ | yahoo.com | $-2.82 \times 10^{-3}$ | conta.cc | $-1.08 \times 10^{-2}$ |
| tumblr.com | $-2.33 \times 10^{-3}$ | nbcnews.com | $1.81 \times 10^{-3}$ | politico.com | $-1.08 \times 10^{-2}$ |
| pscp.tv | $1.85 \times 10^{-3}$ | senate.gov | $1.60 \times 10^{-3}$ | senate.gov | $9.19 \times 10^{-3}$ |
| washingtonexam[*].com | $1.82 \times 10^{-3}$ | espn.com | $-1.58 \times 10^{-3}$ | abcnews.com | $-6.90 \times 10^{-3}$ |
| foursquare.com | $-1.77 \times 10^{-3}$ | techchrunch.com | $-1.52 \times 10^{-3}$ | reddit.com | $-6.53 \times 10^{-3}$ |
| furnishhome.ru | $-1.71 \times 10^{-3}$ | change.org | $1.47 \times 10^{-3}$ | github.com | $-6.22 \times 10^{-3}$ |
| house.gov | $-1.65 \times 10^{-3}$ | ebay.com | $-1.39 \times 10^{-3}$ | mailchimp.com | $-5.98 \times 10^{-3}$ |
| wordpress.com | $-1.55 \times 10^{-3}$ | foursquare.com | $-1.21 \times 10^{-3}$ | wsj.com | $-5.95 \times 10^{-3}$ |
| washingtonpost.com | $-1.53 \times 10^{-3}$ | usatoday.com | $-1.18 \times 10^{-3}$ | wabcradio.com | $-5.86 \times 10^{-3}$ |
| yahoo.com | $-1.53 \times 10^{-3}$ | nationalreview.com | $9.91 \times 10^{-4}$ | instagram.com | $-5.78 \times 10^{-3}$ |
| fllwrs.com | $-1.34 \times 10^{-3}$ | washingtonpost.com | $-9.65 \times 10^{-4}$ | hubs.ly | $-5.59 \times 10^{-3}$ |
| twittascope.com | $1.32 \times 10^{-3}$ | smotrim.ru | $-9.56 \times 10^{-4}$ | foxnews.com | $-5.54 \times 10^{-3}$ |
| rawstory.com | $-1.28 \times 10^{-3}$ | sba.gov | $9.19 \times 10^{-4}$ | msn.com | $-5.39 \times 10^{-3}$ |

**(A2): Chn(19) $\rightarrow$ Chn(19) / Chn(20)**

| domain | IG(val.) | domain | IG(test) | domain | IG(base.) |
|---|---|---|---|---|---|
| cnn.com | $-2.88 \times 10^{-3}$ | justunfollow.com | $5.48 \times 10^{-3}$ | nytimes.com | $-2.08 \times 10^{-2}$ |
| washingtonpost.com | $-2.49 \times 10^{-3}$ | etsy.com | $-7.58 \times 10^{-4}$ | abouthub.info | $-1.30 \times 10^{-2}$ |
| cdt.org | $2.16 \times 10^{-3}$ | tumblr.com | $-5.40 \times 10^{-4}$ | conta.cc | $-1.08 \times 10^{-2}$ |
| wordpress.com | $-1.63 \times 10^{-3}$ | yahoo.com | $-4.56 \times 10^{-4}$ | politico.com | $-1.08 \times 10^{-2}$ |
| wsj.com | $-1.59 \times 10^{-3}$ | cnn.com | $-4.53 \times 10^{-4}$ | senate.gov | $9.19 \times 10^{-3}$ |
| medium.com | $-1.11 \times 10^{-3}$ | foursquare.com | $-3.83 \times 10^{-4}$ | abcnews.com | $-6.90 \times 10^{-3}$ |
| bloomberg.com | $9.32 \times 10^{-4}$ | house.gov | $-3.57 \times 10^{-4}$ | reddit.com | $-6.53 \times 10^{-3}$ |
| eventbrite.com | $-8.78 \times 10^{-4}$ | medium.com | $-3.50 \times 10^{-4}$ | github.com | $-6.22 \times 10^{-3}$ |
| theconversation.com | $7.66 \times 10^{-4}$ | apnews.com | $2.57 \times 10^{-4}$ | mailchimp.com | $-5.98 \times 10^{-3}$ |
| yahoo.com | $-7.61 \times 10^{-4}$ | conservativej[*].com | $2.54 \times 10^{-4}$ | wsj.com | $-5.95 \times 10^{-3}$ |
| ap.org | $7.39 \times 10^{-4}$ | twittascope.com | $2.25 \times 10^{-4}$ | wabcradio.com | $-5.86 \times 10^{-3}$ |
| inewsource.org | $6.83 \times 10^{-4}$ | sba.gov | $2.12 \times 10^{-4}$ | instagram.com | $-5.78 \times 10^{-3}$ |
| www.gov.uk | $-6.53 \times 10^{-4}$ | theconversation.com | $-1.62 \times 10^{-4}$ | hubs.ly | $-5.59 \times 10^{-3}$ |
| mktw.net | $-6.42 \times 10^{-4}$ | wired.com | $-1.36 \times 10^{-4}$ | foxnews.com | $-5.54 \times 10^{-3}$ |
| sen.gov | $-6.28 \times 10^{-4}$ | github.com | $-1.29 \times 10^{-4}$ | msn.com | $-5.39 \times 10^{-3}$ |

**(A3): Iran(19) $\rightarrow$ Iran(19) / Iran(21)**

| domain | IG(val.) | domain | IG(test) | domain | IG(base.) |
|---|---|---|---|---|---|
| huffpost.com | $-4.25 \times 10^{-3}$ | untappd.com | $2.41 \times 10^{-2}$ | nytimes.com | $-1.83 \times 10^{-2}$ |
| foursquare.com | $-3.89 \times 10^{-3}$ | gofundme.com | $1.87 \times 10^{-2}$ | abouthub.info | $-1.12 \times 10^{-2}$ |
| medium.com | $-3.68 \times 10^{-3}$ | twittascope.com | $-1.34 \times 10^{-2}$ | politico.com | $-1.00 \times 10^{-2}$ |
| untappd.com | $3.58 \times 10^{-3}$ | bbc.co.uk | $1.24 \times 10^{-2}$ | conta.cc | $-9.88 \times 10^{-3}$ |
| cnn.com | $-3.09 \times 10^{-3}$ | wired.com | $-9.16 \times 10^{-3}$ | senate.gov | $9.27 \times 10^{-3}$ |
| senate.gov | $2.82 \times 10^{-3}$ | iheart.com | $8.79 \times 10^{-3}$ | abcnews.com | $-6.67 \times 10^{-3}$ |
| house.gov | $-2.66 \times 10^{-3}$ | npr.org | $8.12 \times 10^{-3}$ | reddit.com | $-5.96 \times 10^{-3}$ |
| etsy.com | $-2.41 \times 10^{-3}$ | senate.gov | $7.63 \times 10^{-3}$ | wsj.com | $-5.94 \times 10^{-3}$ |
| apple.news | $-2.34 \times 10^{-3}$ | washingtonexaminer.com | $6.55 \times 10^{-3}$ | github.com | $-5.58 \times 10^{-3}$ |
| economist.com | $2.19 \times 10^{-3}$ | house.gov | $-6.23 \times 10^{-3}$ | mailchimp.com | $-5.53 \times 10^{-3}$ |
| abouthub.info | $-2.14 \times 10^{-3}$ | justunfollow.com | $6.04 \times 10^{-3}$ | wabcradio.com | $-5.32 \times 10^{-3}$ |
| yahoo.com | $-2.13 \times 10^{-3}$ | wordpress.com | $-5.87 \times 10^{-3}$ | hubs.ly | $-5.23 \times 10^{-3}$ |
| amazon.com | $-2.05 \times 10^{-3}$ | tumblr.com | $-5.75 \times 10^{-3}$ | instagram.com | $-5.16 \times 10^{-3}$ |
| twittascope.com | $-1.79 \times 10^{-3}$ | economist.com | $5.54 \times 10^{-3}$ | foxnews.com | $-5.10 \times 10^{-3}$ |
| tumblr.com | $-1.73 \times 10^{-3}$ | bloomberg.com | $5.01 \times 10^{-3}$ | msn.com | $-4.86 \times 10^{-3}$ |

**(B1): Chn(19) + Iran(19) → Rus(18) / Rus(20)**

| domain | IG(val.) | domain | IG(test) | domain | IG(base.) |
|---|---|---|---|---|---|
| nbcnews.com | $3.28 \times 10^{-3}$ | inc.com | $-7.94 \times 10^{-3}$ | nytimes.com | $-2.07 \times 10^{-2}$ |
| tumblr.com | $-2.85 \times 10^{-3}$ | yahoo.com | $-3.76 \times 10^{-3}$ | abouthub.info | $-1.22 \times 10^{-2}$ |
| house.gov | $-2.78 \times 10^{-3}$ | nbcnews.com | $2.32 \times 10^{-3}$ | politico.com | $-1.09 \times 10^{-2}$ |
| senate.gov | $2.50 \times 10^{-3}$ | foursquare.com | $-1.88 \times 10^{-3}$ | conta.cc | $-1.08 \times 10^{-2}$ |
| pscp.tv | $2.41 \times 10^{-3}$ | soundcloud.com | $1.69 \times 10^{-3}$ | senate.gov | $8.49 \times 10^{-3}$ |
| foursquare.com | $-2.16 \times 10^{-3}$ | espn.com | $-1.34 \times 10^{-3}$ | abcnews.com | $-7.85 \times 10^{-3}$ |
| wordpress.com | $-2.05 \times 10^{-3}$ | usatoday.com | $-1.16 \times 10^{-3}$ | reddit.com | $-6.85 \times 10^{-3}$ |
| thehill.com | $-2.01 \times 10^{-3}$ | washingtonpost.com | $-1.15 \times 10^{-3}$ | mailchimp.com | $-6.82 \times 10^{-3}$ |
| washingtonpost.com | $-1.95 \times 10^{-3}$ | change.org | $1.11 \times 10^{-3}$ | wsj.com | $-6.40 \times 10^{-3}$ |
| furnishhome.ru | $-1.84 \times 10^{-3}$ | apnews.com | $1.06 \times 10^{-3}$ | github.com | $-6.19 \times 10^{-3}$ |
| bbc.co.uk | $1.51 \times 10^{-3}$ | house.gov | $-1.05 \times 10^{-3}$ | wabcradio.com | $-6.12 \times 10^{-3}$ |
| forbes.com | $-1.44 \times 10^{-3}$ | sba.gov | $9.44 \times 10^{-4}$ | hubs.ly | $-5.82 \times 10^{-3}$ |
| yahoo.com | $-1.41 \times 10^{-3}$ | senate.gov | $9.17 \times 10^{-4}$ | foxnews.com | $-5.82 \times 10^{-3}$ |
| bglradio.net | $-1.41 \times 10^{-3}$ | pinterest.com | $-8.24 \times 10^{-4}$ | instagram.com | $-5.75 \times 10^{-3}$ |
| fllwrs.com | $-1.36 \times 10^{-3}$ | huffpost.com | $-7.92 \times 10^{-4}$ | msn.com | $-5.26 \times 10^{-3}$ |

**(B2): Rus(18) + Iran(19) → Chn(19) / Chn(20)**

| domain | IG(val.) | domain | IG(test) | domain | IG(base.) |
|---|---|---|---|---|---|
| washingtonpost.com | $-2.20 \times 10^{-3}$ | justunfollow.com | $6.73 \times 10^{-3}$ | nytimes.com | $-1.85 \times 10^{-2}$ |
| cnn.com | $-2.02 \times 10^{-3}$ | tumblr.com | $-6.54 \times 10^{-4}$ | abouthub.info | $-1.06 \times 10^{-2}$ |
| twittascope.com | $-1.56 \times 10^{-3}$ | etsy.com | $-5.16 \times 10^{-4}$ | conta.cc | $-1.04 \times 10^{-2}$ |
| wordpress.com | $-1.09 \times 10^{-3}$ | yahoo.com | $-5.03 \times 10^{-4}$ | politico.com | $-1.00 \times 10^{-2}$ |
| senate.gov | $1.04 \times 10^{-3}$ | cnn.com | $-4.89 \times 10^{-4}$ | senate.gov | $8.92 \times 10^{-3}$ |
| cdt.org | $9.19 \times 10^{-4}$ | foursquare.com | $-4.69 \times 10^{-4}$ | abcnews.com | $-7.24 \times 10^{-3}$ |
| yahoo.com | $-8.57 \times 10^{-4}$ | house.gov | $-3.58 \times 10^{-4}$ | reddit.com | $-6.16 \times 10^{-3}$ |
| sen.gov | $-8.55 \times 10^{-4}$ | conservativej[*].com | $3.32 \times 10^{-4}$ | wsj.com | $-5.92 \times 10^{-3}$ |
| theconversation.com | $8.21 \times 10^{-4}$ | medium.com | $-2.83 \times 10^{-4}$ | mailchimp.com | $-5.55 \times 10^{-3}$ |
| medium.com | $-7.94 \times 10^{-4}$ | theconversation.com | $-2.27 \times 10^{-4}$ | wabcradio.com | $-5.50 \times 10^{-3}$ |
| epsomguardian.co.uk | $7.85 \times 10^{-4}$ | apnews.com | $2.06 \times 10^{-4}$ | hubs.ly | $-5.48 \times 10^{-3}$ |
| msn.com | $-7.39 \times 10^{-4}$ | github.com | $-1.98 \times 10^{-4}$ | instagram.com | $-5.29 \times 10^{-3}$ |
| bloomberg.com | $7.12 \times 10^{-4}$ | wired.com | $-1.94 \times 10^{-4}$ | foxnews.com | $-5.28 \times 10^{-3}$ |
| furnishhome.ru | $-6.89 \times 10^{-4}$ | sba.gov | $1.66 \times 10^{-4}$ | msn.com | $-5.02 \times 10^{-3}$ |
| inewsource.org | $6.69 \times 10^{-4}$ | cnsnews.com | $-1.49 \times 10^{-4}$ | github.com | $-4.80 \times 10^{-3}$ |

**(B3): Chn(19) + Rus(18) → Iran(19) / Iran(21)**

| domain | IG(val.) | domain | IG(test) | domain | IG(base.) |
|---|---|---|---|---|---|
| foursquare.com | $-4.15 \times 10^{-3}$ | gofundme.com | $1.80 \times 10^{-2}$ | nytimes.com | $-1.95 \times 10^{-2}$ |
| cnn.com | $-3.91 \times 10^{-3}$ | bbc.co.uk | $1.28 \times 10^{-2}$ | conta.cc | $-1.12 \times 10^{-2}$ |
| house.gov | $-3.85 \times 10^{-3}$ | twittascope.com | $-9.93 \times 10^{-3}$ | abouthub.info | $-1.09 \times 10^{-2}$ |
| medium.com | $-3.66 \times 10^{-3}$ | house.gov | $-9.60 \times 10^{-3}$ | politico.com | $-1.04 \times 10^{-2}$ |
| huffpost.com | $-3.38 \times 10^{-3}$ | iheart.com | $7.84 \times 10^{-3}$ | senate.gov | $9.25 \times 10^{-3}$ |
| senate.gov | $2.67 \times 10^{-3}$ | wordpress.com | $-7.82 \times 10^{-3}$ | abcnews.com | $-7.66 \times 10^{-3}$ |
| apple.news | $-2.44 \times 10^{-3}$ | npr.org | $7.49 \times 10^{-3}$ | reddit.com | $-6.26 \times 10^{-3}$ |
| amazon.com | $-2.10 \times 10^{-3}$ | cnn.com | $-6.56 \times 10^{-3}$ | wsj.com | $-5.96 \times 10^{-3}$ |
| greenlifestylech[*].com | $1.84 \times 10^{-3}$ | washingtonex[*].com | $6.13 \times 10^{-3}$ | github.com | $-5.93 \times 10^{-3}$ |
| tumblr.com | $-1.70 \times 10^{-3}$ | senate.gov | $6.10 \times 10^{-3}$ | mailchimp.com | $-5.86 \times 10^{-3}$ |
| usa.gov | $-1.63 \times 10^{-3}$ | justunfollow.com | $5.54 \times 10^{-3}$ | wabcradio.com | $-5.82 \times 10^{-3}$ |
| economist.com | $1.62 \times 10^{-3}$ | bloomberg.com | $5.52 \times 10^{-3}$ | instagram.com | $-5.67 \times 10^{-3}$ |
| theguardian.com | $-1.51 \times 10^{-3}$ | tumblr.com | $-5.47 \times 10^{-3}$ | foxnews.com | $-5.65 \times 10^{-3}$ |
| yahoo.com | $-1.46 \times 10^{-3}$ | wired.com | $-5.17 \times 10^{-3}$ | hubs.ly | $-5.40 \times 10^{-3}$ |
| conta.cc | $-1.37 \times 10^{-3}$ | economist.com | $4.89 \times 10^{-3}$ | msn.com | $-5.10 \times 10^{-3}$ |

**Table 5.** IG values for individual domains in each trial and val, test, and baseline set. For long domain names, [*] denotes truncation.

## E Frequent and Removed domains

Here we show the most frequent domains which are retained or censored in each dataset. We specifically show the result at the most stringent threshold $\gamma_{max} = 0.4$ where MLP still demonstrates strong performance.

# Baseline

| **indicator** | counts | **indicator** | tf-idf | **censored** ($\gamma_{max}$=0.4) | counts |
|---|---|---|---|---|---|
| UNCOMMON | 770187 | instagram.com | 1464.24 | twitter.com | 1571276 |
| instagram.com | 283505 | house.gov | 511.7 | youtube.com | 211489 |
| twittascope.com | 91120 | fllwrs.com | 477.48 | facebook.com | 106082 |
| house.gov | 68485 | soundcloud.com | 417.33 | vine.co | 25835 |
| nytimes.com | 58666 | twittascope.com | 415.91 | ift.tt | 22096 |
| washingtonpost.com | 37356 | nytimes.com | 380.88 | google.com | 17822 |
| senate.gov | 28874 | foxnews.com | 289.61 | blogspot.com | 16239 |
| usa.gov | 20972 | espn.com | 288.08 | cnn.it | 8579 |
| theguardian.com | 20216 | washingtonpost.com | 286.39 | reuters.com | 8203 |
| huffpost.com | 19937 | pscp.tv | 281.8 | bbc.in | 8193 |
| wsj.com | 19904 | github.com | 250.51 | hill.cm | 7066 |
| foxnews.com | 19632 | www.gov.uk | 240.47 | fxn.ws | 6111 |
| tumblr.com | 19555 | wordpress.com | 239.23 | breitbart.com | 5917 |
| pinterest.com | 19031 | apple.com | 236.77 | chinanews.com | 5753 |
| bloomberg.com | 18553 | twitch.tv | 220.57 | telegraph.co.uk | 5287 |
| foursquare.com | 18350 | huffpost.com | 219.8 | twimg.com | 5157 |
| wordpress.com | 17313 | tumblr.com | 217.38 | vimeo.com | 5028 |
| soundcloud.com | 17177 | birdops.com | 210.94 | nypost.com | 5007 |
| fllwrs.com | 16862 | snpy.tv | 210.9 | etsy.com | 4062 |
| apnews.com | 16724 | foursquare.com | 207.91 | dailycaller.com | 4038 |
| swarmapp.com | 15441 | amazon.com | 206.87 | kp.ru | 3865 |
| amazon.com | 14282 | theguardian.com | 196.59 | spoti.fi | 3850 |
| cnn.com | 14275 | bbc.co.uk | 195.18 | independent.co.uk | 3839 |
| pscp.tv | 13428 | atmlb.com | 193.08 | unfollowspy.com | 3734 |
| usatoday.com | 12696 | gofundme.com | 181.99 | t.me | 3641 |
| politico.com | 12322 | linkedin.com | 175.83 | vk.com | 3636 |
| thehill.com | 12002 | uapp.ly | 171.29 | livejournal.com | 3621 |
| medium.com | 11073 | curiouscat.me | 169.83 | bbc.com | 3406 |
| etsy.com | 9797 | pinterest.com | 166.41 | spotify.com | 3340 |
| bbc.co.uk | 9624 | buzzfeed.com | 165.26 | dailymail.co.uk | 3222 |
| npr.org | 9299 | usa.gov | 164.36 | newsweek.com | 3189 |
| forbes.com | 8892 | conta.cc | 160.76 | ruposters.ru | 3170 |
| cnbc.com | 8800 | senate.gov | 159.12 | rusnovosti.ru | 3147 |
| linkedin.com | 8664 | medium.com | 157.13 | periodismodeportivoin[*].com | 3121 |
| apple.com | 8524 | eventbrite.com | 150.9 | twitpic.com | 3070 |
| github.com | 8477 | ble.ac | 143.32 | regnum.ru | 2855 |
| ebay.com | 8190 | wsj.com | 143.09 | smarturl.it | 2812 |
| eventbrite.com | 8082 | cnn.com | 139.38 | lat.ms | 2597 |
| crowdfireapp.com | 8039 | hudl.com | 133.94 | cbsloc.al | 2577 |
| untappd.com | 8009 | politico.com | 128.7 | rbc.ru | 2425 |
| iacr.org | 7715 | usatoday.com | 126.92 | dld.bz | 2423 |
| yahoo.com | 6787 | forbes.com | 126.03 | yfrog.com | 2344 |
| latimes.com | 6756 | iacr.org | 122.46 | washex.am | 2300 |
| conta.cc | 6554 | crowdfireapp.com | 122.11 | read.bi | 2197 |
| cbsnews.com | 6546 | thehill.com | 121.6 | soompi.com | 2192 |
| buzzfeed.com | 6486 | change.org | 121.29 | chicagotribune.com | 2182 |
| apple.news | 6427 | untappd.com | 121.19 | washingtontimes.com | 2177 |
| www.gov.uk | 6349 | nfl.com | 117.88 | theblaze.com | 2176 |
| time.com | 5862 | npr.org | 114.22 | mash.to | 2031 |
| abouthub.info | 5761 | abcnews.com | 114.22 | conscores.org | 2010 |
| vox.com | 5596 | apple.news | 109.49 | miamiherald.com | 2002 |
| flipit.com | 5560 | bible.com | 109.38 | sfgate.com | 1895 |

# Russia (2018)

| indicator | counts | indicator | tf-idf | censored ($\gamma_{max}$=0.4) | counts |
|---|---|---|---|---|---|
| UNCOMMON | 101869 | instagram.com | 501.11 | livejournal.com | 617933 |
| instagram.com | 27361 | cnn.com | 481.74 | riafan.ru | 452984 |
| washingtonpost.com | 16305 | wikipedia.org | 263.78 | twitter.com | 285859 |
| foxnews.com | 12065 | oprf.ru | 257.69 | ria.ru | 166088 |
| nytimes.com | 6143 | sdelanounas.ru | 216.52 | yandex.ru | 152580 |
| usatoday.com | 4831 | swarmapp.com | 180.14 | gazeta.ru | 129518 |
| huffpost.com | 4520 | dni.ru | 137.09 | youtube.com | 122115 |
| theguardian.com | 4220 | nytimes.com | 118.84 | rt.com | 84782 |
| wordpress.com | 3965 | rvns.co | 108.87 | ift.tt | 64237 |
| tumblr.com | 3242 | ukraina.ru | 99.21 | nevnov.ru | 63584 |
| soundcloud.com | 3190 | tumblr.com | 98.1 | vesti.ru | 52729 |
| politico.com | 2769 | iz.ru | 84.76 | kievsmi.net | 50567 |
| cnn.com | 2529 | crowdfireapp.com | 57.37 | vk.com | 45196 |
| apnews.com | 2315 | theguardian.com | 56.06 | kiev-news.com | 44616 |
| wsj.com | 2306 | wordpress.com | 54.68 | inforeactor.ru | 39339 |
| rawstory.com | 1892 | huffpost.com | 53.88 | championat.com | 35713 |
| bloomberg.com | 1867 | whitehouse.gov | 48.24 | lenta.ru | 34658 |
| thehill.com | 1805 | apple.com | 47.39 | tass.ru | 33355 |
| pscp.tv | 1634 | change.org | 46.88 | emaidan.com.ua | 33270 |
| washingtonexaminer.com | 1589 | snpy.tv | 44.71 | vine.co | 25509 |
| abcnews.com | 1563 | soundcloud.com | 43.95 | vk.cc | 23394 |
| wikipedia.org | 1504 | washingtonpost.com | 42.22 | lifenews.ru | 23009 |
| amazon.com | 1432 | sky.com | 40.49 | rbc.ru | 22143 |
| buzzfeed.com | 1391 | foxnews.com | 39.41 | podrobnosti.biz | 21969 |
| nbcnews.to | 1384 | afp.com | 39.19 | nahnews.com.ua | 21049 |
| freebeacon.com | 1321 | bbc.co.uk | 39.09 | izvestia.ru | 20644 |
| townhall.com | 1285 | wsj.com | 38.62 | fontanka.ru | 20545 |
| time.com | 1264 | golos-dnr.ru | 35.71 | e1.ru | 20142 |
| swarmapp.com | 1218 | amazon.com | 32.78 | exerciseworkout.pw | 17285 |
| yahoo.com | 1185 | usatoday.com | 29.25 | mr-7.ru | 17228 |
| medium.com | 1057 | rusnext.ru | 27.76 | losefattips.pw | 16805 |
| apple.com | 1009 | salon.com | 23.24 | facebook.com | 16364 |
| cbsnews.com | 991 | espn.com | 22.94 | breitbart.com | 16292 |
| vice.com | 979 | pscp.tv | 22.73 | twib.in | 16089 |
| latimes.com | 882 | msn.com | 22.29 | cbslocal.com | 15810 |
| nationalreview.com | 830 | rawstory.com | 21.05 | om1.ru | 15372 |
| snpy.tv | 782 | nbcnews.to | 20.51 | prokazan.ru | 14887 |
| dailywire.com | 745 | vice.com | 18.93 | inosmi.ru | 14757 |
| motherjones.com | 679 | sumall.com | 18.38 | msk.ru | 14590 |
| npr.org | 672 | politico.com | 18.36 | ksnt.com | 14572 |
| shareaholic.com | 671 | buzzfeed.com | 18.19 | dailym.ai | 14435 |
| usa.gov | 663 | bloomberg.com | 18.17 | tvrain.ru | 14293 |
| sdelanounas.ru | 647 | time.com | 17.77 | 161.ru | 13696 |
| msnbc.com | 626 | iheart.com | 17.55 | newsnn.ru | 13608 |
| slate.me | 608 | freebeacon.com | 17.09 | trkterra.ru | 13468 |
| apple.news | 604 | apnews.com | 16.67 | meduza.io | 13455 |
| bbc.co.uk | 593 | uapp.ly | 16.66 | sport-express.ru | 13349 |
| redstate.com | 589 | healthcare.gov | 16.47 | 74.ru | 13024 |
| thedailybeast.com | 586 | businessinsider.com | 15.61 | burnfat.pw | 12865 |
| lifenews.com | 575 | aka.ms | 15.27 | exercisequote.pw | 12396 |
| twittascope.com | 549 | economist.com | 15.03 | newinform.com | 12350 |
| cnbc.com | 541 | atmlb.com | 14.67 | losefat.pw | 11831 |

# China (2019)

| indicator | counts |
|---|---|
| UNCOMMON | 131091 |
| instagram.com | 17516 |
| nytimes.com | 8756 |
| tumblr.com | 6757 |
| soundcloud.com | 5850 |
| bbc.co.uk | 3153 |
| uapp.ly | 2514 |
| wordpress.com | 1902 |
| forbes.com | 1824 |
| etsy.com | 1720 |
| latimes.com | 1598 |
| pscp.tv | 1486 |
| amazon.com | 1205 |
| huffpost.com | 1109 |
| espn.com | 1044 |
| twitch.tv | 990 |
| fllwrs.com | 906 |
| apple.com | 878 |
| wsj.com | 848 |
| cnn.com | 818 |
| crowdfireapp.com | 741 |
| foursquare.com | 666 |
| tcrn.ch | 656 |
| foxnews.com | 617 |
| datpiff.com | 605 |
| yahoo.com | 569 |
| theguardian.com | 539 |
| engadget.com | 532 |
| curiouscat.me | 523 |
| gigam.es | 492 |
| feedly.com | 398 |
| twittascope.com | 396 |
| washingtonpost.com | 383 |
| typepad.com | 379 |
| swarmapp.com | 345 |
| justunfollow.com | 338 |
| pinterest.com | 328 |
| pulse.me | 327 |
| military.com | 297 |
| mashable.com | 290 |
| linkedin.com | 287 |
| flickr.com | 274 |
| nba.com | 262 |
| patch.com | 257 |
| bloomberg.com | 246 |
| microsoft.com | 233 |
| natgeo.com | 226 |
| justgiving.com | 224 |
| usatoday.com | 224 |
| usa.gov | 212 |
| foodandwine.com | 205 |
| thinkprogress.org | 197 |

| indicator | tf-idf |
|---|---|
| instagram.com | 110.62 |
| forbes.com | 101.06 |
| wsj.com | 83.31 |
| nba.com | 74.34 |
| pscp.tv | 57.7 |
| twitch.tv | 49.74 |
| tumblr.com | 45.29 |
| uapp.ly | 43.4 |
| nytimes.com | 42.68 |
| curiouscat.me | 39.99 |
| time.com | 39.77 |
| soundcloud.com | 34.44 |
| natgeo.com | 34.39 |
| apple.com | 33.13 |
| amazon.com | 30.43 |
| app.link | 27.99 |
| scmp.com | 25.93 |
| reddit.com | 25.87 |
| wordpress.com | 23.63 |
| amazonaws.com | 23.25 |
| bbc.co.uk | 22.29 |
| fllwrs.com | 20.95 |
| foodandwine.com | 20.42 |
| crowdfireapp.com | 19.81 |
| variety.com | 18.58 |
| onlyfans.com | 18.12 |
| cnn.com | 17.64 |
| yahoo.com | 17.48 |
| justunfollow.com | 17.4 |
| ew.com | 16.95 |
| pinterest.com | 16.09 |
| politico.com | 15.68 |
| younow.com | 15.43 |
| flickr.com | 15.26 |
| linkfire.com | 14.85 |
| espn.com | 14.37 |
| fiverr.com | 14.09 |
| etsy.com | 13.74 |
| theguardian.com | 13.05 |
| cbsnews.com | 12.99 |
| shareaholic.com | 12.79 |
| cntraveler.com | 12.54 |
| foursquare.com | 12.32 |
| facebook.com | 12.06 |
| medium.com | 11.75 |
| ebay.com | 11.33 |
| nbcsports.com | 10.98 |
| eonline.com | 10.71 |
| bleacherreport.com | 10.7 |
| pagesix.com | 10.48 |
| theglobeandmail.com | 10.46 |
| washingtonpost.com | 10.44 |

| censored ($\gamma_{max}$=0.4) | counts |
|---|---|
| twitter.com | 526189 |
| dld.bz | 195629 |
| youtube.com | 137703 |
| feedburner.com | 115155 |
| miss50percent.de | 107453 |
| happymuslimfamily.net | 62821 |
| ift.tt | 52636 |
| blogspot.com | 43738 |
| tandl.me | 43348 |
| telkomsel.com | 19472 |
| mychinanews.com | 19221 |
| google.com | 19212 |
| favstar.fm | 18991 |
| tsel.me | 18721 |
| iphonehacks.com | 18097 |
| facebook.com | 18028 |
| hampersbylucy.co.uk | 16229 |
| entrepreneur.com | 15844 |
| g1entretenimento.com | 15543 |
| bbc.in | 12567 |
| kom.ps | 12233 |
| tistory.com | 11974 |
| rol.co.id | 11776 |
| curiosidadeeinformacao.com | 11416 |
| chinanews.com | 11414 |
| feedsportal.com | 11266 |
| blingbling.guru | 11177 |
| na-ss.com | 10750 |
| ask.fm | 10293 |
| twitpic.com | 10112 |
| herokuapp.com | 9163 |
| seocheckout.com | 9084 |
| cgtn.com | 9033 |
| okezone.com | 8969 |
| noticiasboa.com | 8420 |
| mejorsaludybelleza.com | 8304 |
| ithome.com | 7987 |
| osversos.com | 7909 |
| nicovideo.jp | 7254 |
| antonioarzola.net | 7057 |
| thingstodoinleicester.com | 6607 |
| segurosdecochebaratos101.com | 6354 |
| top-domains.ch | 6167 |
| sinaimg.cn | 5917 |
| sina.com.cn | 5664 |
| dlcconcepts.com | 5513 |
| envoque.news | 5374 |
| aplitrak.com | 4638 |
| ecns.cn | 4533 |
| roundteam.co | 4498 |
| tmi.me | 4495 |
| tuguchis.mx | 4384 |

# Iran (2019)

| indicator | counts | indicator | tf-idf | censored ($\gamma_{max}$=0.4) | counts |
|---|---|---|---|---|---|
| UNCOMMON | 511760 | instagram.com | 242.26 | awdnews.com | 332374 |
| instagram.com | 13943 | theguardian.com | 184.91 | twitter.com | 241386 |
| theguardian.com | 2506 | pscp.tv | 158.72 | irib.ir | 217793 |
| amazon.com | 1980 | amazon.com | 97.03 | parstoday.com | 153488 |
| nytimes.com | 1977 | mirror.co.uk | 95.83 | tel-avivtimes.com | 143601 |
| wordpress.com | 1768 | reddit.com | 92.89 | countdown2040.com | 139262 |
| huffpost.com | 1602 | soundcloud.com | 87.84 | nilenetonline.com | 113892 |
| bbc.co.uk | 1368 | huffpost.com | 75.28 | youtube.com | 97822 |
| reddit.com | 1223 | wikipedia.org | 73.22 | sahartv.ir | 68003 |
| pscp.tv | 1221 | nytimes.com | 73.2 | ift.tt | 61936 |
| washingtonpost.com | 1155 | wordpress.com | 72.01 | whatsupic.com | 56026 |
| cnn.com | 1140 | ebay.com | 68.85 | hugedomains.com | 53051 |
| bloomberg.com | 1077 | bbc.co.uk | 67.95 | 7sabah.com | 40922 |
| fllwrs.com | 909 | cnn.com | 57.24 | blogspot.com | 37202 |
| npr.org | 883 | change.org | 53.08 | iuvmpress.com | 37175 |
| pinterest.com | 863 | bloomberg.com | 50.72 | 7sabah.com.tr | 36348 |
| ebay.com | 826 | washingtonpost.com | 47.99 | facebook.com | 31935 |
| soundcloud.com | 824 | fllwrs.com | 47.48 | rt.com | 30039 |
| cnbc.com | 673 | pinterest.com | 45.27 | libertyfrontpress.com | 26177 |
| rawstory.com | 570 | swarmapp.com | 44.21 | al-hadath24.com | 25096 |
| yahoo.com | 508 | medium.com | 43.87 | beritadunia.net | 24965 |
| mirror.co.uk | 483 | npr.org | 43.29 | realnienovosti.com | 24529 |
| oregonlive.com | 470 | oregonlive.com | 41.59 | hindkhabar.in | 23440 |
| politico.com | 459 | rawstory.com | 38.14 | sachtimes.com | 23102 |
| etsy.com | 448 | buffer.com | 37.78 | alwaght.com | 18792 |
| usatoday.com | 411 | altnews.in | 37.41 | whatthebeep.in | 16635 |
| change.org | 404 | etsy.com | 36.85 | islamtimes.org | 15430 |
| forbes.com | 373 | yahoo.com | 34.42 | iuvmonline.com | 15004 |
| medium.com | 332 | wef.ch | 33.9 | alwasatnews.com | 14972 |
| apple.com | 327 | metro.co.uk | 33.5 | jordan-times.com | 14943 |
| wikipedia.org | 326 | apnews.com | 33.33 | hindkhabar.com | 12604 |
| wsj.com | 324 | afp.com | 28.55 | hourriya-tagheer.org | 12354 |
| salon.com | 297 | ebay.to | 26.87 | theroot.com | 12111 |
| vice.com | 296 | cnbc.com | 25.59 | qudspal.com | 11930 |
| economist.com | 293 | sky.com | 25.35 | documentinterdit.com | 11816 |
| ebay.to | 263 | sumall.com | 25.23 | nthnews.net | 11690 |
| apnews.com | 254 | politico.com | 24.88 | ptv.io | 11540 |
| tumblr.com | 252 | cpix.me | 24.49 | telegram.me | 8909 |
| thinkprogress.org | 231 | theintercept.com | 24.15 | jamnews.ir | 8302 |
| sky.com | 231 | thehill.com | 23.77 | yedinot.com | 8167 |
| vox.com | 228 | salon.com | 23.67 | al-saudia.net | 8143 |
| foxnews.com | 228 | huffingtonpost.co.uk | 22.06 | alwaienews.net | 7902 |
| thehill.com | 222 | apple.com | 21.29 | wilayat.in | 6908 |
| nbcnews.com | 218 | google.co.uk | 20.12 | tahreerparty.net | 5955 |
| scientificamerican.com | 217 | thetimes.co.uk | 20.04 | reportaseislam.com | 5842 |
| time.com | 212 | ynetnews.com | 19.95 | alkawthartv.com | 5535 |
| cbsnews.com | 209 | economist.com | 19.44 | realiran.org | 5504 |
| ft.com | 208 | scientificamerican.com | 19.31 | toonsonline.net | 5195 |
| talkingpointsmemo.com | 200 | thinkprogress.org | 19.15 | pakonlinenews.com | 5176 |
| go.com | 199 | go.com | 18.68 | isna.ir | 5084 |
| abcnews.com | 195 | irishtimes.com | 18.54 | presstv.ir | 4709 |
| businessinsider.com | 188 | vice.com | 18.42 | mdn.tv | 4638 |

# Russia (2020)

| indicator | counts |
|---|---|
| UNCOMMON | 33522 |
| instagram.com | 22190 |
| foursquare.com | 7586 |
| swarmapp.com | 2104 |
| furnishhome.ru | 1151 |
| iz.ru | 615 |
| dni.ru | 552 |
| ft.com | 532 |
| crowdfireapp.com | 521 |
| tumblr.com | 518 |
| nytimes.com | 467 |
| wsj.com | 405 |
| sdelanounas.ru | 373 |
| wordpress.com | 365 |
| change.org | 329 |
| sumall.com | 252 |
| theguardian.com | 236 |
| linkedin.com | 227 |
| apple.com | 206 |
| medium.com | 203 |
| washingtonpost.com | 197 |
| wikipedia.org | 181 |
| csmonitor.com | 173 |
| izhgpk.ru | 169 |
| soundcloud.com | 164 |
| forbes.com | 155 |
| fllwrs.com | 149 |
| newsomsk.ru | 134 |
| huffpost.com | 122 |
| gizmodo.com | 101 |
| thenextweb.com | 88 |
| nike.com | 87 |
| awe.sm | 74 |
| bloomberg.com | 72 |
| bbc.co.uk | 71 |
| pscp.tv | 71 |
| cnn.com | 56 |
| thinkprogress.org | 54 |
| ustream.tv | 53 |
| anekdot.ru | 52 |
| time.com | 51 |
| diletant.media | 45 |
| thediplomat.com | 42 |
| foreignpolicy.com | 41 |
| motherjones.com | 40 |
| whitehouse.gov | 39 |
| valuemytweets.com | 36 |
| oprf.ru | 36 |
| buzzfeed.com | 35 |
| theverge.com | 35 |
| quora.com | 34 |
| thehill.com | 33 |

| indicator | tf-idf |
|---|---|
| iz.ru | 293.69 |
| dni.ru | 193.44 |
| instagram.com | 144.6 |
| quora.com | 126.54 |
| thenextweb.com | 115.03 |
| furnishhome.ru | 107.5 |
| foursquare.com | 78.0 |
| change.org | 61.06 |
| anekdot.ru | 57.35 |
| apple.com | 30.15 |
| diletant.media | 29.41 |
| tumblr.com | 22.09 |
| finanz.ru | 19.72 |
| pscp.tv | 18.61 |
| wordpress.com | 18.02 |
| wikipedia.org | 16.82 |
| buzzfeed.com | 15.44 |
| medium.com | 14.77 |
| crowdfireapp.com | 14.53 |
| smotrim.ru | 14.33 |
| sdelanounas.ru | 12.61 |
| forbes.com | 10.57 |
| oprf.ru | 10.12 |
| swarmapp.com | 9.91 |
| strana.ua | 9.71 |
| flickr.com | 9.3 |
| elle.com | 8.54 |
| linkfire.com | 8.28 |
| ukraina.ru | 7.66 |
| ustream.tv | 7.21 |
| bbc.co.uk | 7.02 |
| nasa.gov | 6.94 |
| brave.com | 6.71 |
| natgeo.com | 6.7 |
| reddit.com | 6.33 |
| awe.sm | 6.27 |
| izhgpk.ru | 5.15 |
| socialmediatoday.com | 5.12 |
| gotowebinar.com | 4.96 |
| military.com | 4.57 |
| flipit.com | 4.54 |
| tcrn.ch | 4.47 |
| rollingstone.com | 4.38 |
| nytimes.com | 4.37 |
| esquire.com | 4.19 |
| nike.com | 4.08 |
| shareaholic.com | 3.73 |
| slideshare.net | 3.68 |
| whitehouse.gov | 3.64 |
| facebook.com | 3.6 |
| wsj.com | 3.56 |
| sumall.com | 3.48 |

| censored ($\gamma_{max}$=0.4) | counts |
|---|---|
| lenta.ru | 213528 |
| yandex.ru | 176599 |
| rambler.ru | 72433 |
| newkaliningrad.ru | 62448 |
| 161.ru | 42756 |
| profile.ru | 41755 |
| ufa1.ru | 39093 |
| 76.ru | 37515 |
| vk.cc | 29395 |
| ngs24.ru | 28842 |
| twitter.com | 25478 |
| 29.ru | 24533 |
| ngs55.ru | 23252 |
| youtube.com | 22823 |
| ali.pub | 18388 |
| livejournal.com | 17377 |
| er.ru | 15853 |
| dropi.ru | 15011 |
| 45.ru | 10915 |
| mos.ru | 10406 |
| facebook.com | 9954 |
| ria.ru | 9256 |
| kinopoisk.ru | 9084 |
| valdaiclub.com | 8720 |
| life.ru | 6978 |
| filmz.ru | 5855 |
| vk.com | 5114 |
| tass.ru | 5108 |
| mail.ru | 5054 |
| gazeta.ru | 4298 |
| t.me | 3579 |
| auto.ru | 3413 |
| rt.com | 3384 |
| onedio.ru | 3357 |
| onf.ru | 2539 |
| orientalreview.org | 2443 |
| openreporter.ru | 2347 |
| kp.ru | 2287 |
| championat.com | 2230 |
| twitpic.com | 2210 |
| regnum.ru | 2136 |
| ridus.ru | 2120 |
| sports.ru | 2081 |
| antimaidan.ru | 2081 |
| n1.ru | 2080 |
| rbc.ru | 1973 |
| interfax.ru | 1968 |
| lifenews.ru | 1947 |
| ihodl.com | 1945 |
| izvestia.ru | 1860 |
| bbratstvo.com | 1840 |
| uwidata.com | 1705 |

# China (2020)

| indicator | counts |
|---|---|
| UNCOMMON | 1693 |
| axios.com | 231 |
| medium.com | 36 |
| pscp.tv | 31 |
| bloomberg.com | 30 |
| cnn.com | 26 |
| nytimes.com | 24 |
| amazon.com | 21 |
| foxnews.com | 18 |
| linkfire.com | 17 |
| instagram.com | 13 |
| theguardian.com | 10 |
| washingtonpost.com | 10 |
| cbsnews.com | 10 |
| weebly.com | 9 |
| wordpress.com | 9 |
| nu.nl | 9 |
| time.com | 9 |
| usatoday.com | 9 |
| lasvegassun.com | 7 |
| yahoo.com | 7 |
| wsj.com | 6 |
| apple.com | 5 |
| economist.com | 5 |
| app.link | 5 |
| bbc.co.uk | 5 |
| swarmapp.com | 4 |
| change.org | 4 |
| katu.com | 4 |
| rfa.org | 4 |
| broadway.com | 4 |
| natgeo.com | 3 |
| tumblr.com | 3 |
| adobe.com | 3 |
| quora.com | 3 |
| google.com | 3 |
| insider.com | 3 |
| soundcloud.com | 3 |
| variety.com | 3 |
| nikkei.com | 2 |
| diletant.media | 2 |
| wistia.com | 2 |
| obama.org | 2 |
| mediamatters.org | 2 |
| meetedgar.com | 2 |
| marvel.com | 2 |
| sns.mx | 2 |
| people.com | 2 |
| petapixel.com | 2 |
| eonline.com | 2 |
| forbes.com | 2 |
| fiverr.com | 2 |

| indicator | tf-idf |
|---|---|
| axios.com | 164.41 |
| linkfire.com | 29.84 |
| adobe.com | 14.58 |
| bloomberg.com | 13.46 |
| wordpress.com | 10.48 |
| weebly.com | 10.32 |
| medium.com | 10.03 |
| amazon.com | 9.86 |
| pscp.tv | 9.31 |
| people.com | 6.79 |
| fiverr.com | 6.69 |
| onlyfans.com | 6.6 |
| diletant.media | 6.2 |
| change.org | 5.69 |
| app.link | 5.51 |
| apple.com | 5.43 |
| yahoo.com | 5.35 |
| nike.com | 5.27 |
| nytimes.com | 5.2 |
| wsj.com | 4.07 |
| bbc.co.uk | 4.06 |
| cc.com | 4.04 |
| washingtonpost.com | 3.96 |
| standard.co.uk | 3.81 |
| swarmapp.com | 3.67 |
| buzzfeednews.com | 3.66 |
| viewbug.com | 3.42 |
| fanlink.to | 3.27 |
| time.com | 3.08 |
| instagram.com | 3.03 |
| anchor.fm | 2.91 |
| tumblr.com | 2.88 |
| natgeo.com | 2.86 |
| theguardian.com | 2.36 |
| uscis.gov | 2.35 |
| pandora.com | 2.28 |
| cnn.com | 2.1 |
| justice.gov | 2.06 |
| foxnews.com | 2.03 |
| cnet.com | 1.94 |
| lasvegassun.com | 1.82 |
| soundcloud.com | 1.81 |
| variety.com | 1.62 |
| pbs.org | 1.47 |
| eonline.com | 1.32 |
| foreignpolicy.com | 1.25 |
| cbsnews.com | 1.2 |
| apnews.com | 1.12 |
| quora.com | 1.02 |
| usatoday.com | 0.81 |
| economist.com | 0.59 |
| etsy.com | 0.49 |

| censored ($\gamma_{max}$=0.4) | counts |
|---|---|
| youtube.com | 8148 |
| twitter.com | 1130 |
| creaders.net | 829 |
| temaretik.com | 669 |
| rapradar.com | 503 |
| pansci.asia | 348 |
| dwnews.com | 263 |
| russian7.ru | 262 |
| creu.ru | 249 |
| femmie.ru | 248 |
| yandex.ru | 235 |
| temadnya.com | 188 |
| mychinanews.com | 184 |
| facebook.com | 149 |
| cyrillitsa.ru | 146 |
| russian7.ru | 140 |
| back-in-ussr.com | 138 |
| bbc.com | 131 |
| chinanews.com | 122 |
| vseonauke.com | 112 |
| lifehacker.ru | 107 |
| kulturologia.ru | 102 |
| targetplay.ru | 101 |
| dnpmag.com | 99 |
| cgtn.com | 97 |
| interesnosti.com | 92 |
| p2pb2b.io | 90 |
| discuss.com.hk | 85 |
| pics.ru | 79 |
| novate.ru | 79 |
| mistika.xyz | 77 |
| sci-hit.com | 70 |
| greatpicture.ru | 66 |
| blogspot.com | 64 |
| grammy-s.ru | 59 |
| trendymen.ru | 58 |
| omode.info | 51 |
| ift.tt | 51 |
| bet535casinoonline.com | 49 |
| kzg.io | 47 |
| snatchnews.com | 46 |
| disq.us | 45 |
| globaltimes.cn | 44 |
| bonus.express | 40 |
| klikabol.com | 40 |
| cluber.com.ua | 38 |
| everve.net | 38 |
| mirvokrugnas.com | 37 |
| adme.ru | 35 |
| tothemoon.game | 34 |
| promoidom.com | 33 |
| medpravila.com | 32 |

# Iran (2021)

| indicator | counts |
|---|---|
| UNCOMMON | 1338 |
| nytimes.com | 373 |
| cbsnews.com | 213 |
| theguardian.com | 208 |
| wsj.com | 203 |
| washingtonpost.com | 186 |
| cnn.com | 186 |
| instagram.com | 171 |
| rawstory.com | 148 |
| pscp.tv | 111 |
| thehill.com | 97 |
| politico.com | 76 |
| businessinsider.com | 69 |
| huffpost.com | 64 |
| foxnews.com | 59 |
| nbcnews.com | 55 |
| usatoday.com | 53 |
| thedailybeast.com | 52 |
| msn.com | 45 |
| forbes.com | 44 |
| economist.com | 39 |
| time.com | 35 |
| yahoo.com | 33 |
| grahamcluley.com | 31 |
| abcnews.com | 30 |
| npr.org | 28 |
| vox.com | 27 |
| cnbc.com | 26 |
| axios.com | 26 |
| clevelandclinic.com | 26 |
| apnews.com | 26 |
| msnbc.com | 24 |
| latimes.com | 23 |
| hillreporter.com | 23 |
| slate.com | 22 |
| change.org | 21 |
| bloomberg.com | 20 |
| theatlantic.com | 20 |
| medium.com | 18 |
| salon.com | 18 |
| mediaite.com | 17 |
| motherjones.com | 16 |
| dailykos.com | 16 |
| theweek.com | 15 |
| thenationalpulse.com | 15 |
| petrescuereport.com | 14 |
| whitehouse.gov | 13 |
| sky.com | 13 |
| go.com | 12 |
| actblue.com | 12 |
| ft.com | 12 |
| newyorker.com | 11 |

| indicator | tf-idf |
|---|---|
| nytimes.com | 19.49 |
| wsj.com | 18.16 |
| instagram.com | 10.53 |
| theguardian.com | 9.81 |
| rawstory.com | 9.2 |
| whitehouse.gov | 7.52 |
| pscp.tv | 7.21 |
| cnn.com | 6.01 |
| usatoday.com | 5.79 |
| washingtonpost.com | 5.07 |
| forbes.com | 4.12 |
| thehill.com | 4.07 |
| dailywire.com | 3.98 |
| theverge.com | 3.62 |
| flipit.com | 3.61 |
| msn.com | 3.44 |
| theconversation.com | 3.25 |
| latimes.com | 3.18 |
| businessinsider.com | 3.15 |
| mediaite.com | 3.1 |
| politico.com | 3.02 |
| change.org | 2.98 |
| abcnews.com | 2.89 |
| nbcnews.com | 2.82 |
| cbsnews.com | 2.8 |
| wordpress.com | 2.76 |
| sfchronicle.com | 2.63 |
| thepetitionsite.com | 2.38 |
| soundcloud.com | 2.19 |
| theweek.com | 2.06 |
| amazon.com | 2.0 |
| sky.com | 1.91 |
| substack.com | 1.9 |
| variety.com | 1.9 |
| oregonlive.com | 1.89 |
| axios.com | 1.87 |
| thedailybeast.com | 1.85 |
| realclearpolitics.com | 1.82 |
| huffpost.com | 1.82 |
| theatlantic.com | 1.69 |
| time.com | 1.58 |
| apnews.com | 1.57 |
| foxnews.com | 1.54 |
| go.com | 1.5 |
| yahoo.com | 1.4 |
| cnbc.com | 1.39 |
| moveon.org | 1.09 |
| ca.gov | 1.08 |
| vox.com | 1.08 |
| msnbc.com | 1.08 |
| medium.com | 1.07 |
| salon.com | 1.05 |

| censored ($\gamma_{max}$=0.4) | counts |
|---|---|
| htv.mx | 98259 |
| hispantv.com | 43193 |
| youtube.com | 35874 |
| twitter.com | 4918 |
| hispantv.ir | 1312 |
| bing.com | 451 |
| facebook.com | 430 |
| beritadunia.net | 344 |
| thatsmags.com | 334 |
| cnn.it | 288 |
| bc.game | 284 |
| thealtworld.com | 272 |
| balkanspost.com | 263 |
| ahtribune.com | 203 |
| peek.link | 180 |
| tiredearth.com | 161 |
| kitco.com | 152 |
| cryptoglobe.com | 144 |
| cointelegraph.com | 125 |
| bbc.com | 103 |
| reuters.com | 102 |
| hispantv.net | 99 |
| newsweek.com | 89 |
| caitlinjohnstone.com | 89 |
| independent.co.uk | 86 |
| hill.cm | 84 |
| liputan6.com | 78 |
| paulcraigroberts.org | 78 |
| farsnews.ir | 76 |
| blabber.buzz | 73 |
| whatsapp.com | 59 |
| detik.com | 54 |
| nu.or.id | 50 |
| digitalfinancenews.com | 49 |
| aljazeera.com | 49 |
| rt.com | 43 |
| google.com | 42 |
| oal.lu | 41 |
| ift.tt | 41 |
| sindonews.com | 40 |
| altcoinss.com | 40 |
| t.me | 40 |
| politicususa.com | 40 |
| haaretz.com | 40 |
| tradermeetscoder.com | 39 |
| bracingviews.com | 39 |
| hrw.org | 39 |
| dailymail.co.uk | 35 |
| fair.org | 34 |
| daysofpalestine.com | 30 |
| theblaze.com | 27 |
| ibtimes.com | 27 |
